# Identification of the Hub Genes Involved in Stem Cell Treatment for Intervertebral Disc Degeneration: A Conjoint Analysis of Single-Cell and Machine Learning

**DOI:** 10.1155/2023/7055264

**Published:** 2023-01-24

**Authors:** Jianfeng Chen, Fuwei Qi, Guanshen Li, Qiaosong Deng, Chenlin Zhang, Xiaojun Li, Yafeng Zhang

**Affiliations:** ^1^Department of Spine, Wuxi Hospital Affiliated to Nanjing University of Chinese Medicine, Wuxi 214000, China; ^2^Department of Anesthesiology, The First People's Hospital of Taicang, Taicang Affiliated Hospital of Soochow University, Jiangsu Province, 215413 Suzhou City, China; ^3^Department of Orthopaedics, Qidong Hospital of Chinese Medicine, Nantong, Jiangsu 226200, China

## Abstract

Intervertebral disc degeneration (IDD), which is distinguished by a variety of pathologic alterations, is the major cause of low back pain (LBP). Nonetheless, preventative measures or therapies that may delay IDD are scarcely available. In this study, we sought to identify new diagnostic biological markers for IDD. In this first-of-a-kind study combining machine learning, stem cell treatment samples and single-cell sequencing data were collected. Differentially expressed genes (DEGs) were detected from the treatment group and clusters. To filter potential markers, support vector machine analysis and LASSO were performed. LAPTM5 was found to be the hub gene for IDD. In addition, the results of single-cell sequencing demonstrated the critical function of stem cells in IDD. Finally, we found that aging is significantly associated with the rate of stem cells. In general, our results may offer fresh insights that may be used in the investigation of innovative markers for diagnosing IDD. The critical genes identified by the machine learning algorithm could provide new perspectives on IDD.

## 1. Introduction

Intervertebral disc degeneration (IDD) leads to low back pain (LBP), which places a significant burden on individuals and society alike [1]. Chronic low back pain is a major feature of spinal degenerative diseases, the prevalence of which has substantially increased with the burgeoning aging population. Spinal degenerative disease negatively impacts patients' quality of life and intensifies the financial load on their families and society as a whole [2]. In addition to lumbar disc herniation and discogenic low back pain, degenerative disc diseases (DDDs) result from intervertebral disc degeneration [3]. Currently, most patients obtain pain relief from rest or other forms of conservative therapy, in addition to receiving steroids, local anesthetic, and other blocking medications [4]. When other treatment options fail to alleviate symptoms and enhance the quality of life, clinicians often turn to surgical procedures as the last resort. Even though surgery can alleviate pain, it is not capable of replacing the lost nucleus pulposus (NP) cells or reversing the pathologic condition of the intervertebral disc (IVD). Furthermore, surgery is associated with the risk of intraoperative and postoperative complications.

Studies in both preclinical and clinical settings have tested a variety of advanced and novel treatments for degenerative IVD, including the use of mesenchymal stem cells (MSCs), gene transfection, biologics, and growth factors [5]. The capacity of cell-based therapy, both autologous and allogeneic, to target a wide variety of pathways that contribute to IVDD is one reason it is becoming an increasingly popular treatment option. Nonetheless, many obstacles need to be overcome to achieve a satisfactory survival rate in transplanted cells and attain control of cell viability and regulation of differentiation [6].

Currently, MSCs are widely regarded as the most desirable and viable cell source for IVD regeneration, regardless of whether the process takes place *in vitro* or *in vivo* [7]. Most recently, numerous preclinical investigations and clinical trials have shown the potential utility of MSCs in the treatment of degenerated discs [8, 9]. Research has shown that MSCs can develop into an NP-like phenotype, which may optimize hydration in the IVD, elevate disc height, and enhance matrix formation of glycosaminoglycans (GAGs) [10]. The coculturing of MSCs with NP cells (NPCs) has also been shown to contribute to bidirectional communication between the MSCs and NPCs. Consequently, transplanted cells can alter NPC functions via the release of bioactive substances, such as anabolic growth factors, in addition to undergoing differentiation and the de novo production of the extracellular matrix (ECM) [11]. Moreover, MSCs have anticatabolic and anti-inflammatory characteristics, both of which have the potential to lower cytokine levels; this leads to modulation of the inflammatory niche, resulting in a native NPCs' phenotype that is healthy and nondegenerative [12].

Stem cells have garnered a great deal of interest from researchers and clinicians alike. The fast-paced advancement of stem cell technologies has enabled their adoption in a variety of fields, including hematology, circulation, and orthopedics, among others [13]. Extensive research on IVD and IDD, including that into their underlying mechanism, has led us to believe that a combination of stem cell technology and therapeutic interventions for IDD can sustain the normal physiological structures and functions of the IVD, in addition to reversing the IDD cascade [14]. The benefit of IDD therapy and subsequent recovery may be substantially enhanced by the organic combination of IVD and stem cell technologies; nevertheless, this approach is still contentious [15]. In this study, we investigate the potential of stem cell treatment for IVD based on microarray and single-cell sequencing, with the goal of offering a new perspective on the treatment of IDD.

## 2. Material and Methods

### 2.1. Data Collection and Processing

The microarray data on stem cell therapy for IVD was acquired from the Gene Expression Omnibus (GEO) database in GSE17782 (https://www.ncbi.nlm.nih.gov/geo/query/acc.cgi?acc=GSE17782). Single-cell RNA sequencing data of purified wild-type rat intervertebral disc (GSE154884) were acquired from the GEO database (http://www.ncbi.nlm.nih.gov/geo/).

### 2.2. Differentially Expressed Gene Identification

To standardize and convert the raw data into expression values, we employed the packages available on the Bioconductor website (http://www.bioconductor.org/). To detect the differentially expressed genes (DEGs) between treatment and control samples, the significance analysis included in the empirical Bayes methods of the “limma” package was utilized [16, 17]. The threshold values of |logFC| > 1 and *P* value < 0.05 were employed for selecting significant DEGs.

### 2.3. Analysis of Functions and Pathway Enrichment

Annotation of the functions and pathways of the overlapping DEGs was achieved by employing clusterProfiler (version 3.14.3) for Gene Ontology (GO) terms and performing Kyoto Encyclopedia of Genes and Genomes (KEGG) analysis, with a backdrop of the most recent GO annotations (version 3.1.0) and KEGG pathway annotations, respectively [18, 19]. The corresponding bar graph and network graph were then generated (http://www.xiantao.love/).

### 2.4. Validation of Hub Genes Based on Machine Learning Models

The least absolute shrinkage and selection operator (LASSO) model is a regressive analytical arithmetic method that employs regularization to improve the accuracy of the predictive process. The “glmnet” package in R was employed to carry out the LASSO analysis to identify the genes linked to the ability to discriminate between treatment and normal samples [20]. Support vector machine (SVM) is a kind of monitored machine learning technology that is utilized widely for classification and regression analysis. To prevent the problem of overfitting, recursive feature elimination was applied for screening genes from the metadata cohort. SVM recursive feature elimination (SVM-RFE) was applied to screen candidate features to determine which gene set had the best discriminative capacity.

### 2.5. Single-Cell RNA Processing

To examine the transcription heterogeneity of cells inside the IVD milieu, the “Seurat” R package was utilized for integration, preprocessing, elimination of batch effect, and reduction of the nonlinear dimension of the datasets [21]. Cells were clustered with the help of the FindCluster tool. To obtain marker genes within clusters, we employed the FindMarkers tool. Automatic annotation of the cells was done with the SingleR program. We conducted pseudotime trajectory analysis using the default configuration parameters of the Monocle 2 program [22].

### 2.6. Construction of Enrichment Analysis and PPI Network

Enrichment and protein-protein interaction (PPI) analyses were both performed on the DEGs after they were imported into the Metascape database (http://metascape.org). To identify network components at tight junctions, the Molecular Complex Detection algorithm 10 was applied, and all interactions from the STRING database that had a combined score of more than 0.187 were employed. Next, the Cytoscape program was applied to display the PPI network for DEGs in each cluster.

## 3. Results

### 3.1. Determination of DEGs in Stem Cell Treatment

**Figure 1:**
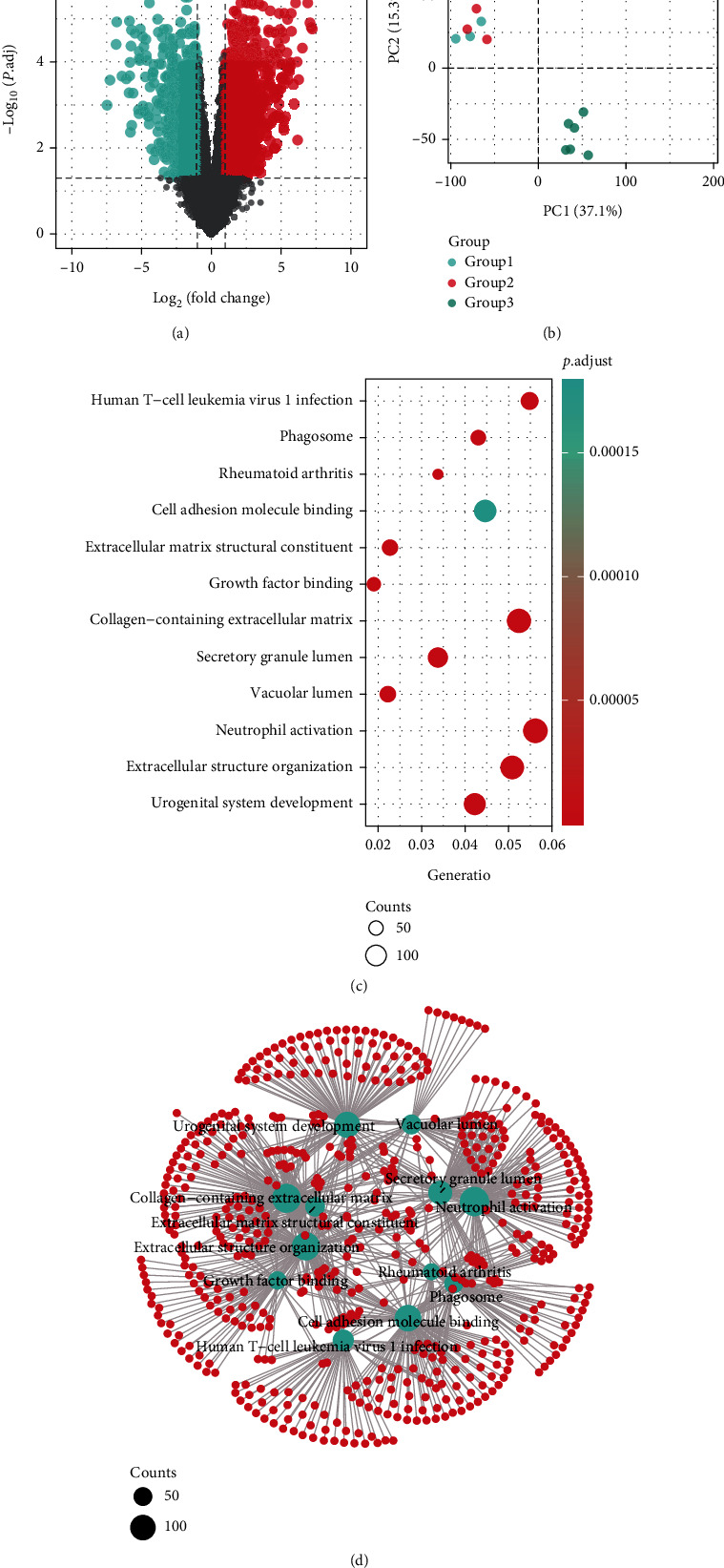
(a) Volcano plot: the fold change and *P* adjusted values were used in the construction of the volcano plot. (b) Principal component analysis of the samples. (c) The pop plot of enrichment analysis of differentially expressed genes. (d) Network of enrichment analysis.

Within the scope of this study, GSE17782 was investigated using a retrospective approach. The “Limma” package was used to investigate the DEGs within the metadata. Initially, 2944 DEGs were identified, as shown in Figure 1(a). A two-dimensional principal component analysis cluster diagram was used to illustrate the gene expression matrix associated with the dataset. The vast majority of samples exhibited considerable clustering, suggesting that the sample source was reliable (Figure 1(b)).

### 3.2. KEGG Pathway Enrichment Analyses of Stem Cell Treated DEGs

KEGG analysis was performed using clusterProfiler to annotate DEGs that overlapped with one another in their functions and pathways. The DEGs were found to be most enriched in human T–cell leukemia virus 1 infection, phagosome, and rheumatoid arthritis pathways (Figures 1(c) and 1(d)).

### 3.3. Determination of Hub Genes

**Figure 2:**
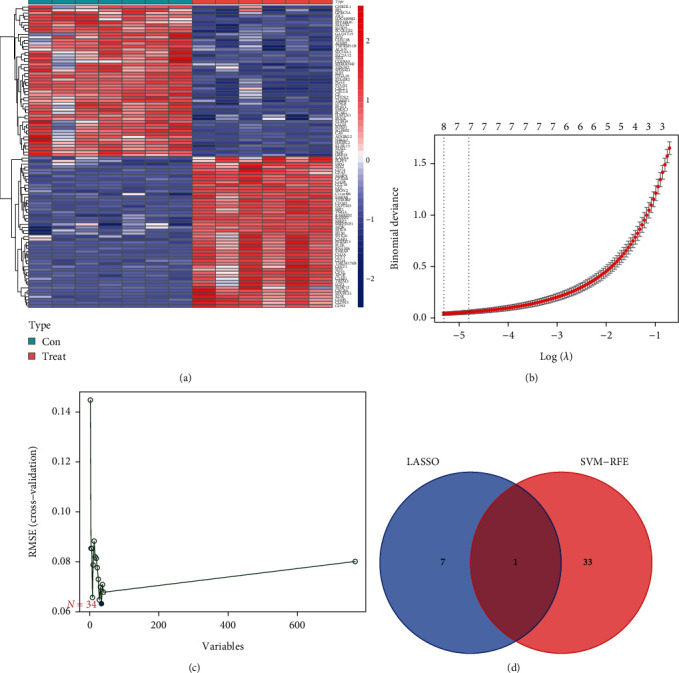
(a) Heatmap of top differentially expressed genes. (b) Number of genes identified using the least absolute shrinkage and selection operator (LASSO) regression method. (c) Number of genes detected using the support vector machine (SVM) regression method. (d) A Venn plot of overlapping genes obtained from LASSO and SVM.

To determine the key biomarkers, two different arithmetic methods were applied.  Figure 2(a) displays the top DEGs that were found. The LASSO method led to the identification of eight variates as hub genes for stem cell therapy (Figure 2(b)). With the aid of the SVM-RFE algorithm, a subset of 34 features shared by the DEGs was discovered (Figure 2(c)). Lastly, the overlapping characteristic, LAPTM5, was selected between the two arithmetic approaches, and this gene may be critical in stem cell treatment (Figure 2(d)).

### 3.4. Single-Cell Sequencing Analysis

**Figure 3:**
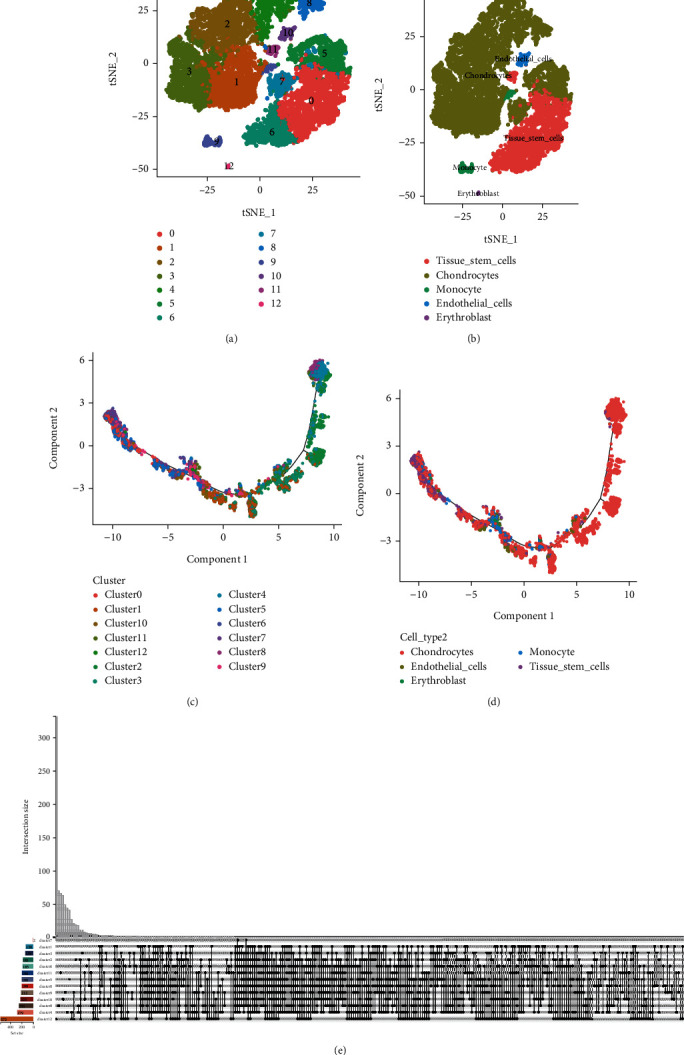
(a, b) Using *t*-distributed stochastic neighbor embedding, a dimensionality reduction algorithm, cells were grouped into 18 kinds, with each hue representing a different annotated phenotype for each cluster. (c, d) Timeline of cells and related cell types. (e) A Venn plot of overlap DEGs in each clusters.

After preprocessing the scRNA-seq data, strict quality control techniques outlined before were used to successfully isolate high-quality cell samples from the disc tissues. Next, to visualize the high-dimensional scRNA-seq data, we applied the t-SNE method to the leading 20 principal components and effectively categorized the cells into 13 different subcategories (Figure 3(a)), which were then annotated to recognize cell types utilizing the “SingleR” R package (Figure 3(b)). After examining the differentiation trajectories and pseudotemporal analysis using the “Monocle2” R package, based on the marker genes found for each cluster, we found it possible to establish a link between these cell clusters and their status (Figures 3(c) and 3(d)). The overlapping genes of DEGs ++ in each cluster are displayed in Figure 3(e).

### 3.5. Gene Enrichment Analysis and PPI Analysis for DEGs in Each Cluster

**Figure 4:**
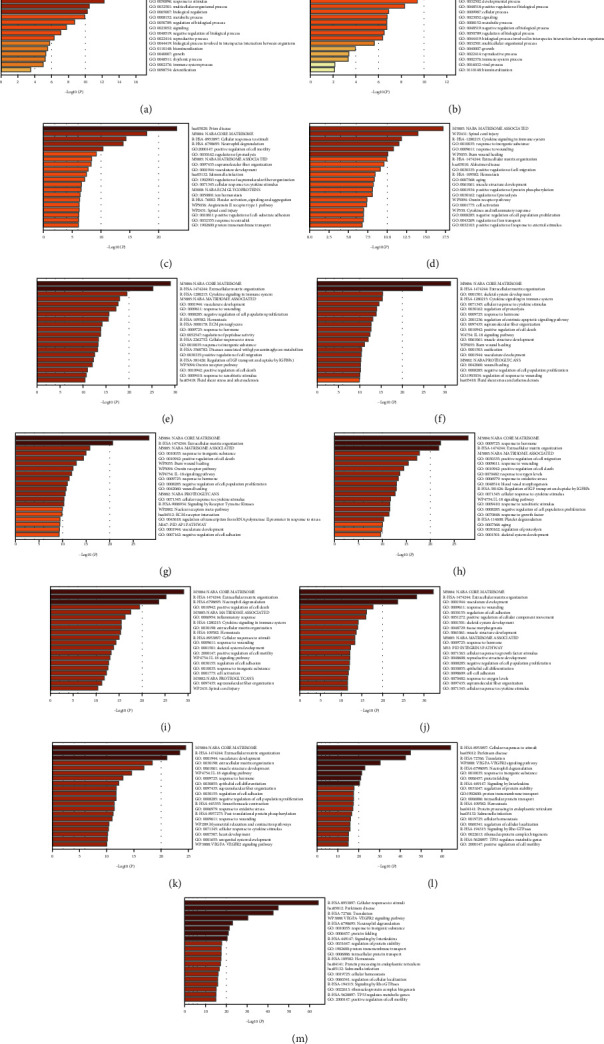
(a–m) Gene Ontology enrichment analysis of each cluster.

**Figure 5:**
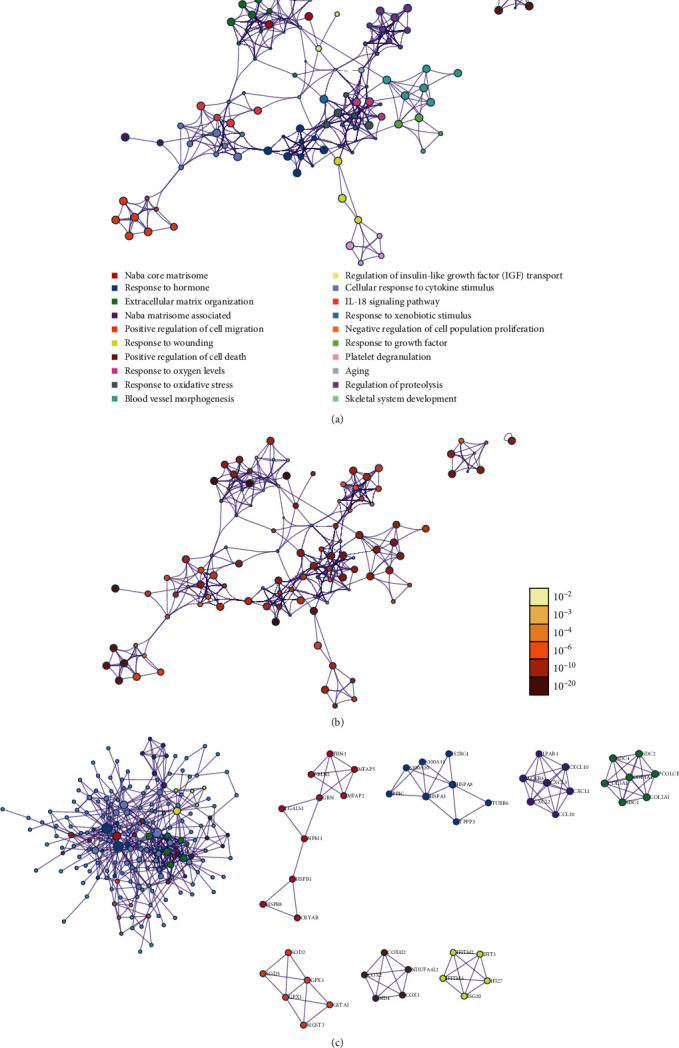
Network of enrichment analysis by (a) pathways and (b) *P* value; protein-protein interaction (PPI) network of differentially expressed genes.

The Metascape database was used to perform functional enrichment analysis on genes that were differentially expressed and methylated, which allowed us to examine the biological activities of these genes. The results for each cluster are presented in Figures 4(a)–4(m). Finally, the enrichment network of cluster 7 is displayed in Figures 5(a) and 5(b). At the same time, the PPI network is depicted in Figure 5(c).

## 4. Discussion

IDD is a degenerative illness that can lead to other conditions, including low back pain and disc herniation. These illnesses have a significant negative impact on the patient's quality of life and impose a significant financial load on society as a whole. Currently, standard therapies are available for IDD in the clinical setting; however, these treatment modalities encounter a “bottleneck,” in that they are unable to reverse the onset and progression of IDD and can only alleviate patients' pain. Treatments found on MSCs and mRNA are prospective targets for IDD therapy, and they are also of considerable use to researchers. The specific molecular mechanisms by which MSCs can be useful for the therapy of IDD are controversial, and clinical studies ought to evaluate their usefulness and safety before being implemented. The effective use of microarrays and bioinformatics has enabled a comprehensive understanding of the molecular processes underlying the onset and progression of illness, which in turn can help determine biological markers and treatment targets.

Although MSCs are currently acknowledged as a high-priority subject in the area of regenerative medicine, they may be difficult to use in the clinical setting owing to storage limitations and the possibility of cell senescence during *in vitro* growth. A recent research study demonstrated that MSCs are capable of secreting exosomes via paracrine signaling and that these exosomes exhibit similar features as those of MSCs. Exosomes generated from MSCs have been shown to exhibit excellent repairability, lower cytotoxicity, and lower immunogenicity. Given that the intervertebral disc is the largest avascular organ in the body, it derives nutrition by rather unique means. The metabolic and nutritional products are transported by blood vessels that are located outside the disc. Nutrients that are transported by capillaries and nutrient canals must first pass through dense hyaline cartilage endplate (CEP) to reach the disc matrix. Additionally, MSCs are created at a nanoscale, which makes them easily transportable and storable. As a result, their potential uses in therapeutic settings are rapidly expanding. Presently, both autologous and allogeneic bone marrow stromal cells (BMSCs) have shown outstanding therapeutic efficacy. Despite the fact that autologous BMSCs have greater homogeneity, cheaper treatment costs, broader applicability, and superior therapeutic potential, allogeneic BMSCs continue to be the cells of choice in stem cell treatment. However, the process of acquiring BMSCs involves invasive procedures; the sample size of clinical studies has been small, and the long-term effectiveness is uncertain; thus, more studies are required.

Chondrocytes undergo terminal differentiation under hypertrophic conditions, which occurs throughout the process of cartilage growth. The hypertrophy-like alterations in chondrocytes are generally accepted to be involved in osteoarthritis. Furthermore, hypertrophic degeneration has been revealed to occur in the NP throughout the course of DDD. As a result, we analyzed the cell fate to evaluate the differentiation statuses of chondrocytes in CEP tissues. In addition, with the use of machine learning, we discovered that LAPTM5 is the hub gene for IDD. This was achieved by stem cell therapy. A recent research report discovered that RUNX2 transactivated the expression of the LAPTM5 gene, which was in turn implicated in the translocation of RANKL. These results confirmed that RUNX2 could modulate osteoclast differentiation via lysosome-related genes that mediate RANKL transport in bone cells. The current findings pointed to the existence of a potential coupling mechanism between osteogenesis and osteoclastogenesis [23]. During the differentiation process of chondrocytes, homeostatic and stem cells were present in the beginning, but stem cells were not present at the end of this process. Through this phenomenon, we speculate that stem cell therapy will be an important means to delay the aging of the lumbar intervertebral disc. To examine the transcriptional heterogeneity of cells in IVD environment, we obtained 13 cell clusters and 4 cell subtypes using single-cell analysis based on GSE154884 dataset. We also use the interaction network of the Cytoscape program cluster 7.

However, our study is mainly based on bioinformatics and relies on public databases, so there are some limitations. First, the patient data is completely from the open database and has not been verified by clinical experiments. Second, the sample size is small, which may limit the statistical power. Therefore, more sample size studies are needed to validate our study in the future.

## 5. Conclusion

To conclude, we provided the first integrated assessment of scRNA-seq and machine learning in the field of IDD. In the diagnosis and treatment of IDD, having a full grasp of cell heterogeneity as well as stem cell treatment might help establish innovative insights and techniques.

## Data Availability

The data can be available via GEO database. More information can be accessed from the corresponding authors.
